# Retraction of “circ_0062491 alleviates periodontitis via the miR-142-5p/IGF1 axis”

**DOI:** 10.1515/med-2023-0888

**Published:** 2023-12-31

**Authors:** Chunlin Wang, Junxia Gong, Dai Li, Xianghui Xing

**Affiliations:** Department of Pediatric Dentistry, Nanjing Stomatological Hospital, Medical School of Nanjing University, Nanjing, Jiangsu 210000, China; Department of First Outpatient, Nanjing Stomatological Hospital, Medical School of Nanjing University, Nanjing, China; Department of Pediatric Dentistry, Nanjing Stomatological Hospital, Medical School of Nanjing University, No. 30, Zhongyang Road, Xuanwu District, Nanjing, Jiangsu 210000, China

Retraction of: Wang C, Gong J, Li D, Xing X. circ_0062491 alleviates periodontitis via the miR-142-5p/IGF1 axis. Open Medicine 2022;17(1):638–647, DOI: 10.1515/med-2022-0442.

This article has been retracted by the publisher due to irregularities in the figures and lack of clarity regarding the origin of the images. As a result, the editors cannot guarantee the legitimacy of the presented data.

